# Structural Integrity of Mechanical Heart Valve Rings During Valve-in-Valve Interventions

**DOI:** 10.1016/j.shj.2026.101118

**Published:** 2026-08-27

**Authors:** Paulina Anna Karlsson, Christian Butter, Anja Haase-Fielitz, Ralf-Uwe Kühnel

**Affiliations:** a Department of Cardiology, University Hospital Heart Center Brandenburg, Brandenburg, Germany; b Faculty of Health Sciences, Brandenburg Medical School (MHB) Theodor Fontane, Neuruppin, Germany; c Institute of Social Medicine and Health System Research, Otto von Guericke University, Magdeburg, Germany; d Department of Cardiac and Vascular Surgery, University Hospital Heart Center Brandenburg, Brandenburg Medical School (MHB) Theodor Fontane, Neuruppin, Germany

**Keywords:** In vitro, Mechanical valve, TAVR, Valve fracture, Valve-in-valve

## Abstract

• Mechanical valve rings fracture only at supraphysiologic pressures.
• Pyrolytic carbon rings show limited expansion and no meaningful diameter gain.
• Structural constraints limit precise prediction of post–valve-in-valve hemodynamics.
• Risk of inadvertent ring damage during leaflet fracture appears low.
• Findings define biomechanical limits of transcatheter valve-in-valve in mechanical heart valves.

## Graphical Abstract

**Figure:**
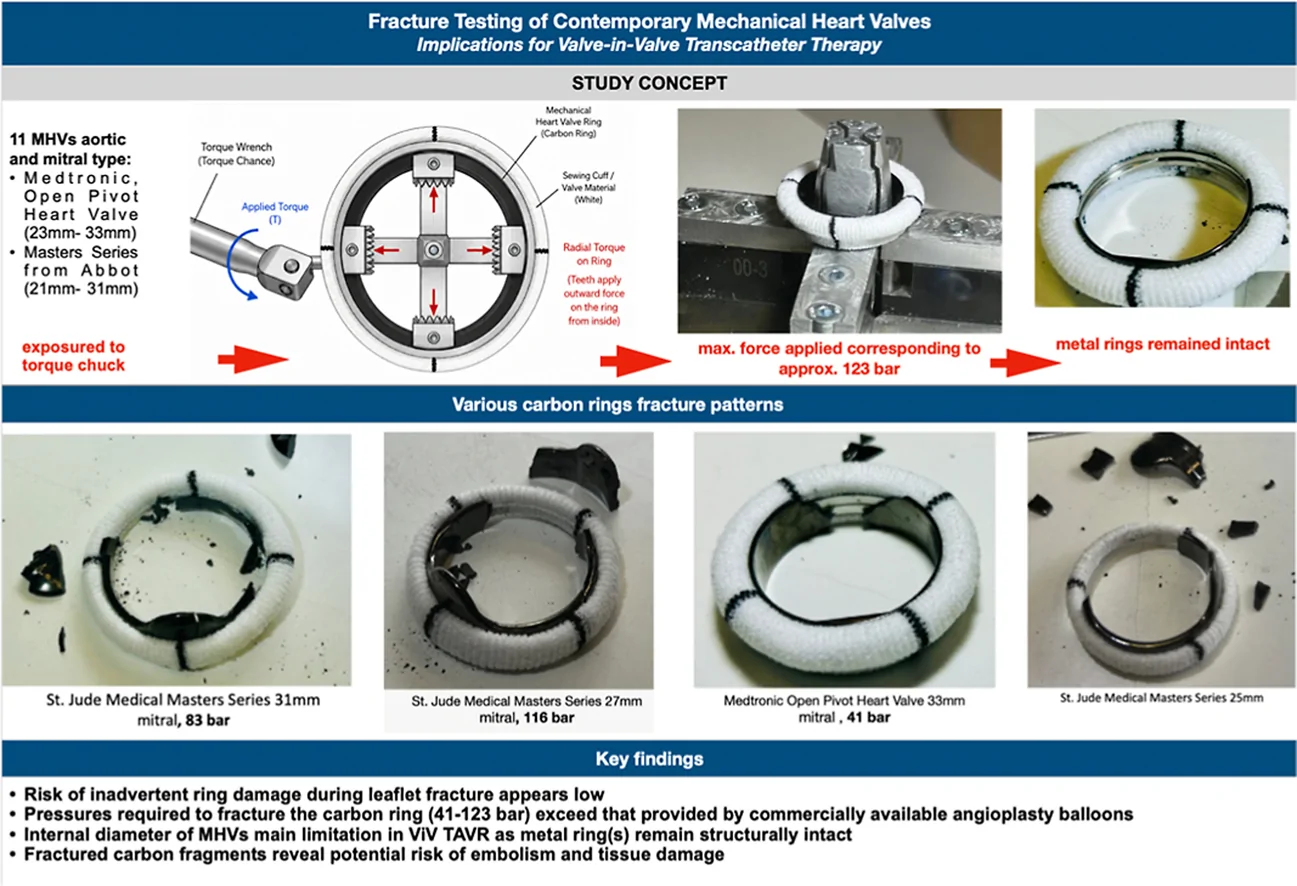

Over the past 2 decades, structural heart interventions have undergone rapid and sustained technological advancement. In contrast, the fundamental design of contemporary mechanical heart valves (MHVs) has remained largely unchanged since their introduction more than half a century ago. This stagnation has been reinforced by the lack of established percutaneous treatment options for prosthetic valve dysfunction, including thrombosis, pannus overgrowth, and structural failure, contributing to a progressive decline in the clinical use of MHVs. However, this paradigm is being challenged by emerging clinical evidence, including successful in-human valve-in-valve (ViV) procedures in MHVs^1^ and demonstrated feasibility of leaflet fracturing with commercially available angioplasty balloons,^2^ suggesting that MHVs may no longer represent an absolute barrier to transcatheter therapy.

Building on evidence from ViV transcatheter aortic valve replacement in failed bioprosthetic valves, in which prosthesis internal diameter strongly influences hemodynamic outcomes, this study investigated whether expansion of MHV rings could overcome similar size-related limitations. To address this aim, we conducted controlled ex vivo fracture testing of 11 contemporary MHVs, providing foundational mechanistic data to inform the development of safe and effective ViV strategies in patients with failed MHVs.

Five Medtronic Open Pivot (aortic and mitral, 23 mm to 33 mm) and 6 Abbott Masters Series (aortic and mitral, 21 mm to 31 mm) MHVs were selected for this study. The valves represented a range of designs and materials commonly used in clinical practice. All valves were visually inspected to confirm structural integrity before testing, after which the mechanical leaflets were carefully removed from all prostheses. This was done intentionally to isolate the mechanical response of the valve ring to radial expansion and eliminate potential effects of leaflet fracture or leaflet-balloon interactions. Consequently, this study was designed to evaluate ring robustness alone. Structural durability testing was performed using a custom-engineered mechanical fixture designed to simulate radial crush conditions during ViV procedures, as illustrated in the Graphical Abstract. The system consisted of 4 high-strength steel jaws arranged in a circular configuration, with a central opening matching the nominal valve diameter. Fracture was defined as the point of visible structural failure. For comparison with clinically established ViV transcatheter aortic valve replacement procedures, fracture torque was converted into an equivalent radial pressure using a simplified geometric model of the valve ring. Assuming a lever arm equal to the effective ring radius, torque was first transformed into radial force and then normalized to the internal surface area of the ring (circumference × height) to obtain an estimated pressure representing an approximation of balloon inflation pressure. Statistical analysis was not performed due to the small sample size and exploratory nature of the study.

Failure pressure varied substantially across valve types and sizes. Among mitral prostheses, failure occurred at 41.1 bar and 123.4 bar for the Medtronic Open Pivot valves (33 mm and 27 mm, respectively) and at 83.0 bar and 116.5 bar for the St. Jude Medical Masters Series valves (31 mm and 27 mm, respectively). Among aortic prostheses, St. Jude Medical Masters Series valves failed at 56.8 bar (25 mm) and 113.2 bar (21 mm). Overall, smaller valve sizes tended to withstand higher pressures before structural failure.

Fracture testing further demonstrated clear material-dependent behavior: carbon-based rings fractured primarily along radial lines with occasionally perpendicular splitting. Microcracking, localized deformation, and fragment rebound or twisting were observed in several cases, raising safety concerns related to embolization or adjacent tissue injury.

Removal of the carbon ring increased the valve diameter by approximately 2 mm and revealed that all tested mechanical valve models contained 1 or more metallic rings. Despite exposure to pressures far exceeding physiological levels, all metallic internal rings remained structurally intact.

Transcatheter ViV procedures in MHVs remain in an early investigational phase but may represent a potential option in highly selected inoperable patients with adequately sized prostheses and in whom favorable outcomes may be achievable without annular dilation. In the present experimental setting, pyrolytic carbon rings only cracked under pressures far exceeding typical balloon inflation pressures, with no meaningful disruption of the underlying metallic framework or significant increase in effective internal diameter.

These structural constraints limit the ability to reliably predict postprocedural hemodynamics—particularly in small prostheses—as current imaging- and registry-based planning tools only provide population-level estimates and cannot precisely model device-specific deformation. Although the structural robustness of MHV rings constrains ViV feasibility by preventing meaningful expansion, it also suggests that the likelihood of inadvertent ring damage during transcatheter leaflet fracture is low.

These findings should not be interpreted as support for routine transcatheter intervention in mechanical valve prostheses, which at present remains limited to highly selected clinical rescue scenarios. The results should be considered in the context of important experimental limitations, including the small number of valves tested and the absence of physiological flow conditions and cyclic fatigue. As such, the results reflect acute mechanical failure under static loading and may not fully capture long-term behavior in vivo. ViV interventions in mechanical prostheses have entered clinical reality; defining their limits may now be as important as demonstrating their feasibility.

## CRediT Authorship Contributions

Paulina Anna Karlsson: (Methodology, Formal Analysis, Writing – Original Draft, Review & Editing), Christian Butter: (Conceptualization, Project Administration, Review & Editing), Anja Haase-Fielitz: (Methodology, Manuscript Revision, Critical Review, Contribution to Study Design), Ralf-Uwe Kühnel: (Supervision, Methodology, Review & Editing).

## Ethics Statement

This study involved in vitro mechanical testing of prosthetic heart valves alone. No human participants, patient data, or animals were involved. Therefore, ethics committee approval and informed consent were not required.

## Funding

The testing of the heart valves was possible thanks to the courtesy of Abbott Laboratories and Medtronic, who provided the 11 mechanical heart valve prostheses for the study. The publication fee was funded by the Brandenburg Medical School publication fund, supported by the Ministry of Science, Research and Cultural Affairs of the State of Brandenburg.

## Disclosure Statement

The authors report no conflict of interest.
